# Prehospital administration of tranexamic acid in trauma patients

**DOI:** 10.1186/s13054-016-1322-5

**Published:** 2016-05-12

**Authors:** Arasch Wafaisade, Rolf Lefering, Bertil Bouillon, Andreas B. Böhmer, Michael Gäßler, Matthias Ruppert, TraumaRegister DGU

**Affiliations:** Department of Trauma and Orthopedic Surgery, University of Witten/Herdecke, Cologne-Merheim Medical Center, Ostmerheimer Strasse 200, D-51109 Cologne, Germany; Institute for Research in Operative Medicine (IFOM), University of Witten/Herdecke, Ostmerheimer Strasse 200, D-51109 Cologne, Germany; Department of Anesthesiology and Intensive Care Medicine, University of Witten/Herdecke, Cologne-Merheim Medical Center, Ostmerheimer Strasse 200, D-51109 Cologne, Germany; Department of Medicine – ADAC Air Rescue Service (Subsidiary of the General German Automobile Club), Munich, Germany

**Keywords:** Trauma, Bleeding, Coagulopathy, Tranexamic acid

## Abstract

**Background:**

Evidence on prehospital administration of the antifibrinolytic tranexamic acid (TXA) in civilian trauma populations is scarce. The aim was to study whether prehospital TXA use in trauma patients was associated with improved outcomes.

**Methods:**

The prehospital database of the ADAC (General German Automobile Club) Air Rescue Service was linked with the TraumaRegister of the German Trauma Society to reidentify patients documented in both registries. Primarily admitted trauma patients (2012 until 2014) who were treated with TXA during the prehospital phase were matched with patients who had not received prehospital TXA, applying propensity score-based matching.

**Results:**

The matching yielded two identical cohorts (*n* = 258 in each group), since there were no significant differences in demographics or injury characteristics (mean Injury Severity Score 24 ± 14 [TXA] vs. 24 ± 16 [control]; *p* = 0.46). The majority had sustained blunt injury (90.3 % vs. 93.0 %; *p* = 0.34). There were no differences with respect to prehospital therapy, including rates of intubation, chest tube insertion or both administration of i.v. fluids and catecholamines. During ER treatment, the TXA cohort received fewer numbers of red blood cells and plasma units, but without reaching statistical significance. Incidences of organ failure, sepsis or thromboembolism showed no significant differences as well, although data were incomplete for these parameters. Early mortality was significantly lower in the TXA group (e.g., 24-h mortality 5.8 % [TXA] vs. 12.4 % [control]; *p* = 0.01), and mean time to death was 8.8 ± 13.4 days vs. 3.6 ± 4.9 days, respectively (*p* = 0.001). Overall hospital mortality was similar in both groups (14.7 % vs. 16.3 %; *p* = 0.72). The most pronounced mortality difference was observed in patients with a high propensity score, reflecting severe injury load.

**Conclusions:**

This is the first civilian study, to our knowledge, in which the effect of prehospital TXA use in trauma patients has been examined. TXA was associated with prolonged time to death and significantly improved early survival. Until further evidence emerges, the results of this study support the use of TXA during prehospital treatment of severely injured patients.

## Background

Exsanguination remains the leading cause of early mortality in trauma patients [1] and recent research has elucidated the role of acute trauma-associated coagulopathy in aggravating haemorrhage [2–4]. Resuscitation strategies for severely injured patients with massive blood loss include several key components, such as transfusion of blood components to reestablish perfusion and coagulation function [5]. Furthermore, several commercially available haemostatic agents are commonly applied as adjuncts to support coagulation [6]. Since clot degradation by early hyperfibrinolysis has been reported to play a major role in traumatic coagulopathy and massive bleeding, recent clinical research has been focused on the antifibrinolytic substance tranexamic acid (TXA) [7]. However, only one large randomised controlled trial—Clinical Randomisation of an Antifibrinolytic in Significant Haemorrhage 2 (CRASH-2)—has examined the effect of in-hospital TXA administration in trauma, but the results have been discussed controversially due to several weaknesses of the trial, such as that the majority of patients were enrolled in developing countries [8, 9]. Altogether, evidence on prehospital TXA use in trauma, especially from European countries, is scarce. Accordingly, existing guidelines either provide no statement or make only a weak recommendation to consider en route administration of TXA [10]. However, since no other drug is approved for coagulation support during prehospital treatment, the aim of the present study was to assess whether prehospital intravenous (i.v.) administration of TXA in trauma patients is associated with improved outcomes.

## Methods

### General German Automobile Club Air Rescue Service database

The General German Automobile Club (ADAC) Air Rescue Service operates 35 air ambulance helicopters throughout Germany and is therefore the largest national provider of Air Rescue Services. For each rescue mission and each patient, information on the prehospital course and treatment is documented including air rescue-specific parameters [11]. The severity of illness and/or injury is assessed by applying the National Advisory Committee for Aeronautics (NACA) score, ranging from I (minor disturbance/injury) to VII (death) [12]. As the equipment for each helicopter regarding medication is regulated according to its respective local policies, TXA has been provided by 20 of the 35 Air Rescue helicopters during the 3-year study period. Patients were treated with TXA at the discretion of the emergency physician.

### TraumaRegister DGU®

The TraumaRegister DGU® (TR-DGU) of the German Trauma Society (Deutsche Gesellschaft für Unfallchirurgie, DGU) was founded in 1993 [13]. The aim of this multicentre database is an anonymous and standardized documentation of severely injured patients. Data are collected prospectively in four consecutive time phases from the site of the accident until discharge from the hospital: (A) prehospital phase, (B) emergency room (ER) and initial surgery, (C) intensive care unit (ICU) and (D) discharge. The documentation includes detailed information on demographics, injury patterns, comorbidities, pre- and in-hospital management, course on the ICU, relevant laboratory findings including data on transfusions, and outcomes of each individual.

The participating hospitals are located primarily in Germany (90 %), but a rising number of hospitals in other countries contribute data as well (e.g., Austria, Belgium, Finland, Luxembourg, Slovenia, Switzerland). Currently, approximately 25,000 cases per year from more than 600 hospitals are entered into the database.

The documentation comprises detailed information including standardized scoring systems (e.g., the Injury Severity Score [ISS]) [14]. All injuries are coded using the Abbreviated Injury Scale [15]. Organ failure was assessed using the Sequential Organ Failure Assessment score [16]. Sepsis was defined according to the Bone criteria, which are close to those of the American College of Chest Physicians/Society of Critical Care Medicine consensus conference definition [17]. The TR-DGU documents the prothrombin time as prothrombin time index (expressed as a percentage of normal; also commonly referred to as *Quick’s test*), where a value of <70 % corresponds to an international normalized ratio >1.3 [3, 18].

### Study population

**Fig. 1:**
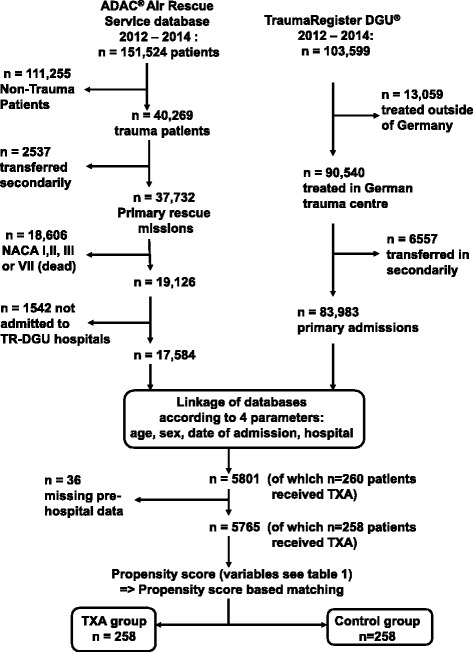
Study outline. *ADAC* General German Automobile Club, *DGU* Deutsche Gesellschaft für Unfallchirurgie, *NACA* National Advisory Committee for Aeronautics, *TR* TraumaRegister, *TXA* tranexamic acid

Due to data security and confidentiality, both databases provide anonymized data; thus, there was no information available about whether individual patients were included in these two independent databases in parallel. As the ADAC Air Rescue Service registry is limited to the prehospital setting without providing information on the patient’s in-hospital course, and since the TR-DGU does not document the prehospital administration of TXA, we linked the data to reidentify patients documented in both registries. Patient records from both databases collected between 1 January 2012 and 31 December 2014 were considered for the present study (Fig. 1).

Patients were included in this study according to the following criteria:

1. ADAC Air Rescue Service database:
  - Primarily admitted trauma patient
  - Critical injury, defined as preclinically assessed NACA IV (potentially life-threatening), NACA V (acute danger) or NACA VI (respiratory and/or cardiac arrest)
  - Admission to a trauma centre participating in the TR-DGU

2. TR-DGU database:
  - Primary admission
  - Treatment in a German trauma centre (i.e., exclusion of trauma centres from other countries)

Data were linked using the parameters age, sex, date and time of injury, and trauma centre (Fig. 1). Thus, a total of 5765 patients were identified as documented in both databases, of whom 258 patients had received i.v. administration of TXA before ER arrival. These 5765 patients were eligible for propensity score-based matched-pairs analysis. A multivariable analysis was conducted using a logistic regression model with prehospital administration of TXA as a dependent variable (Table 1). The resulting independent predictors were applied to calculate a propensity of receiving prehospital TXA. Each of the 258 TXA patients was matched with 1 of the 5507 control patients according to the propensity of receiving TXA; for example, a patient with a propensity of 7 % was matched with another patient with a 7 % propensity. Matching was performed blinded to outcome.

**Table 1:** Multivariable analysis using a logistic regression model with prehospital administration of tranexamic acid as a dependent variable (*n* = 5765)

Variable entered	Regression coefficient β	OR (e^β^; 95 % CI)	*p* Value
Isolated TBI (AIS_head_ ≥3)	−1.009	0.365 (0.165–0.806)	0.013
AIS_thorax_ ≥3	−0.282	0.755 (0.570–0.998)	0.049
AIS_abdomen_ ≥3	0.524	1.689 (1.212–2.355)	0.002
AIS_extremities_ ≥3	0.346	1.414 (1.078–1.854)	0.012
Intubation prehospital	1.043	2.837 (2.022–3.981)	<0.001
Chest tube prehospital	0.506	1.658 (1.093–2.516)	0.017
Penetrating injury	0.369	1.446 (0.970–2.157)	0.070
Prehospital i.v. fluids ≥1000 ml	0.147	1.159 (0.980–1.370)	0.085
Initial systolic blood pressure			
> 110 mmHg	Reference		0.193
81–110 mmHg	−1.164	0.312 (0.074–1.321)	0.114
1–80 mmHg	0.244	1.276 (0.843–1.930)	0.249
0 mmHg	0.141	1.152 (0.849–1.563)	0.364
Age ≥60 years	−0.397	0.672 (0.477–0.947)	0.023
Prehospital GCS score ≤8	−0.166	0.847 (0.630–1.140)	0.275
Constant*	−3.791	0.023	<0.001

*AIS* Abbreviated Injury Scale, *GCS* Glasgow Coma Scale, *i.v.* intravenous, *TBI* traumatic brain injury

The reference category is included in brackets behind the variable name and does not receive a coefficient in the model. ORs have to be interpreted in relation to this standard category

*The constant in a logistic model provides a basic risk that applies to the case that all other coefficients are zero

### Study approval

The medical authorities of the ADAC Air Rescue Service and the scientific committee of the TR-DGU approved the design and publication of this study. This study and publication are in line with the publication guidelines of the TraumaRegisterDGU® and registered as TR-DGU project ID 2015-023. The present study was also approved by the ethics committee of the Faculty of Medicine, University of Witten/Herdecke (Alfred-Herrhausen-Strasse 50, 58448 Witten, Germany; register number 85/2015).

### Statistical analysis

Data were compared between groups using the Mann-Whitney *U* test for continuous variables and Fisher’s exact test for categorical variables, if not indicated otherwise. For comparison of the Kaplan-Meier survival curves, the Mantel-Cox log-rank test was applied. Data are presented as mean with standard deviation (±SD) for continuous variables and as percentages for incidence rates. We applied a significance level *p* < 0.05 to all statistical tests. Statistical analysis was performed using standard statistical software (SPSS version 18.0 software; SPSS, Chicago, IL, USA).

## Results

Among the 5765 patients with complete datasets, 258 pairs with corresponding propensity of TXA administration were identified. As intended, the matching yielded two similar cohorts, as there were no significant differences in demographic data, injury characteristics or prehospital course between treatment groups (Table 2). Patients were predominantly male, and their average ages by group were 43 ± 19 years (TXA group) vs. 41 ± 18 years (control group) (*p* = 0.48). The mean ISSs for the TXA and control groups were 24 ± 14 and 24 ± 16, respectively (*p* = 0.46), and the majority had sustained a blunt injury (90.3 % vs. 93.0 %, respectively; *p* = 0.34). There were also no differences with respect to injury pattern, prehospital vital signs and prehospital therapy, including rates of intubation, chest tube insertion, cardiopulmonary resuscitation and administration of both i.v. fluids and catecholamines. Table 3 summarizes the vital signs and laboratory values upon ER admission, showing no significant differences as well. Before ICU admission, the TXA cohort received fewer numbers of packed red blood cells (pRBC) and plasma units, but without reaching statistical significance. Further haemostatic agents were applied in similar frequencies as well, including fibrinogen concentrate.

**Table 2:** Demographic and prehospital characteristics of trauma patients (2012–2014) with and without tranexamic acid administered during the prehospital phase

	Tranexamic acid (TXA) group (*n* = 258)	Control group (*n* = 258)	*p* Value
Age, years, mean ± SD	43 ± 19	41 ± 18	0.48
Male sex, *n* (%)	187 (72.5)	187 (72.5)	1.00
Traffic accident, *n* (%)	180 (69.8)	189 (73.3)	0.44
Blunt trauma, *n* (%)	233 (90.3)	240 (93.0)	0.34
ISS, points, mean ± SD	24 ± 14	24 ± 16	0.46
AIS_head_ ≥3, *n* (%)	89 (34.5)	95 (36.8)	0.65
AIS_thorax_ ≥3, *n* (%)	120 (46.5)	125 (48.4)	0.72
AIS_abdomen_ ≥3 *n* (%)	54 (20.9)	47 (18.2)	0.51
AIS_extremities_ ≥3, *n* (%)	114 (44.2)	112 (43.4)	0.93
Isolated TBI, AIS_head_ ≥3, *n* (%)	7 (2.7)	8 (3.1)	0.94
SBP at scene ≤90 mmHg, *n* (%)	55 (21.3)	54 (20.9)	1.0
SBP at scene, mmHg, mean ± SD	118 ± 34	116 ± 33	0.36
GCS at scene ≤8, *n* (%)	89 (34.5)	96 (37.2)	0.58
GCS at scene, points, mean ± SD	10.5 ± 4.9	10.2 ± 5.0	0.69
Prehospital treatment			
Intubation, *n* (%)	193 (74.8)	195 (75.6)	0.92
Chest tube insertion, *n* (%)	34 (13.2)	28 (10.9)	0.50
CPR, *n* (%)	11 (4.3)	8 (3.1)	0.64
Catecholamines, *n* (%)	44 (17.1)	50 (19.4)	0.57
i.v. Fluids, ml, mean ± SD	1140 ± 760	1181 ± 919	0.84
Duration of prehospital phase, minutes, mean ± SD	77.2 ± 25.0	74.2 ± 25.1	0.22
Air transport, *n* (%)	210 (81.4)	203 (78.7)	0.51

*AIS* Abbreviated Injury Scale, *CPR* cardiopulmonary resuscitation, *GCS* Glasgow Coma Scale, *i.v.* intravenous, *ISS* Injury Severity Score, *SBP* systolic blood pressure, *SD* standard deviation, *TBI* traumatic brain injury

Data for all 516 patients were documented for the parameters of this table

**Table 3:** Clinical characteristics upon emergency room admission of trauma patients (2012–2014) with and without tranexamic acid administered during the prehospital phase

	Tranexamic acid (TXA) group	Control group	*p* Value
SBP at ER ≤90 mmHg, *n* (%)	51/236 (21.6)	50/247 (20.2)	0.74
SBP at ER, mmHg, mean ± SD (*n*)	114 ± 27 (236)	117 ± 32 (247)	0.19
Haemoglobin,^a^ g/dl, mean ± SD (*n*)	11.7 ± 2.8 (239)	11.4 ± 2.8 (227)	0.33
Base excess,^a^ mmol/L, mean ± SD (*n*)	−3.1 ± 4.7 (221)	−3.5 ± 5.1 (219)	0.38
INR,^a^ mean ± SD (*n*)	1.3 ± 0.7 (230)	1.3 ± 0.5 (216)	0.53
PTI,^a^ Quick’s test points, mean ± SD (*n*)	76 ± 22 (230)	75 ± 23 (216)	0.57
i.v. Fluids,^b^ ml, mean ± SD (*n*)	1945 ± 2889 (182)	1867 ± 2005 (146)	0.46
Crystalloids,^b^ ml, mean ± SD (*n*)	1668 ± 2421 (182)	1523 ± 1817 (146)	0.92
Colloids,^b^ ml, mean ± SD (*n*)	249 ± 591 (182)	317 ± 556 (146)	0.18
pRBC transfusion,^b^ *n* (%)	64/258 (24.8)	65/258 (25.2)	1.00
pRBC units,^b^ mean ± SD (*n*)	1.6 ± 4.3 (258)	2.0 ± 5.8 (258)	0.81
Number of units if given, mean ± SD/median	6.6 ± 6.5/4	7.8 ± 9.4/4	0.81
FFP transfusion,^b^ *n* (%)	40/258 (15.5)	43/258 (16.7)	0.81
FFP units,^b^ mean ± SD (*n*)	1.2 ± 3.8 (258)	1.4 ± 4.6 (258)	0.68
Number of units if given, mean ± SD/median	7.7 ± 6.7/6	8.4 ± 8.1/5	0.68
Platelet transfusion,^b^ *n* (%)	8/258 (3.1)	12/256(4.7)	0.37
Platelet units,^b^ mean ± SD (*n*)	0.1 ± 0.7 (258)	0.1 ± 0.9 (256)	0.37
Number of units if given, mean ± SD/median	3.8 ± 2.1/3.5	2.9 ± 3.0/2	0.37
Massive transfusion,^b^ ≥10 pRBC, *n* (%)	13/258 (5.0)	15/258 (5.8)	0.85
Haemostatic drugs,^b^ *n* (%)	36/205 (17.6)	35/149 (23.5)	0.18
Fibrinogen concentrate,^b^ *n* (%)	26/205 (12.7)	24/149 (16.1)	0.44
Level 1 trauma centre, *n* (%)	182/258 (70.5)	200/258 (77.5)	0.09

*ER* emergency room, *FFP* fresh frozen plasma, *i.v.* intravenous, *INR* international normalized ratio, *pRBC* packed red blood cells, *SD* standard deviation

As some values were missing, the respective population is presented in the denominator for continuous variables and in brackets for categorical variables

^a^Laboratory values measured upon emergency room arrival

^b^pRBC, FFP, intravenous fluids and haemostatic drugs administered between emergency room arrival and intensive care unit admission

The outcome parameters (Table 4) showed no differences with respect to incidence of multiple organ failure, sepsis or thromboembolism, although for these parameters data were not documented for the complete study population.

**Table 4:** Outcome data

	Tranexamic acid (TXA) group	Control group	*p* Value
ICU LOS, days, mean ± SD (*n*)	10.7 ± 12.6 (258)	9.2 ± 11.4 (258)	0.03
Hospital LOS, days, mean ± SD (*n*)	25.5 ± 23.2 (258)	22.3 ± 25.4 (258)	0.04
Thromboembolic event, *n* (%)	4/71 (5.6)	10/121 (8.3)	0.58
Sepsis, *n* (%)	4/67 (6.0)	8/119 (6.7)	1.00
Multiple organ failure, *n* (%)	27/74 (36.5)	35/121 (28.9)	0.34
Time to death, days, mean ± SD (*n*)	8.8 ± 13.4 (258)	3.6 ± 4.9 (258)	0.001
6-h mortality, *n* (%)	5/258 (1.9)	24/258 (9.3)	<0.001
12-h mortality, *n* (%)	9/258 (3.5)	28/258 (10.9)	0.002
24-h mortality, *n* (%)	15/258 (5.8)	32/258 (12.4)	0.01
30-day mortality, *n* (%)	36/258 (14.0)	42/258 (16.3)	0.54
In-hospital mortality overall, *n* (%)	38/258 (14.7)	42/258 (16.3)	0.72
Mortality prognosis in %, based on RISC 2 score (*n*)	15.4 % (258)	15.2 % (258)	0.38

*ICU* intensive care unit, *LOS* length of stay, *RISC* Revised Injury Severity Classification, *SD* standard deviation

As some values were missing, the respective population is documented in brackets for continuous variables and in the denominator for categorical variables

**Fig. 2:**
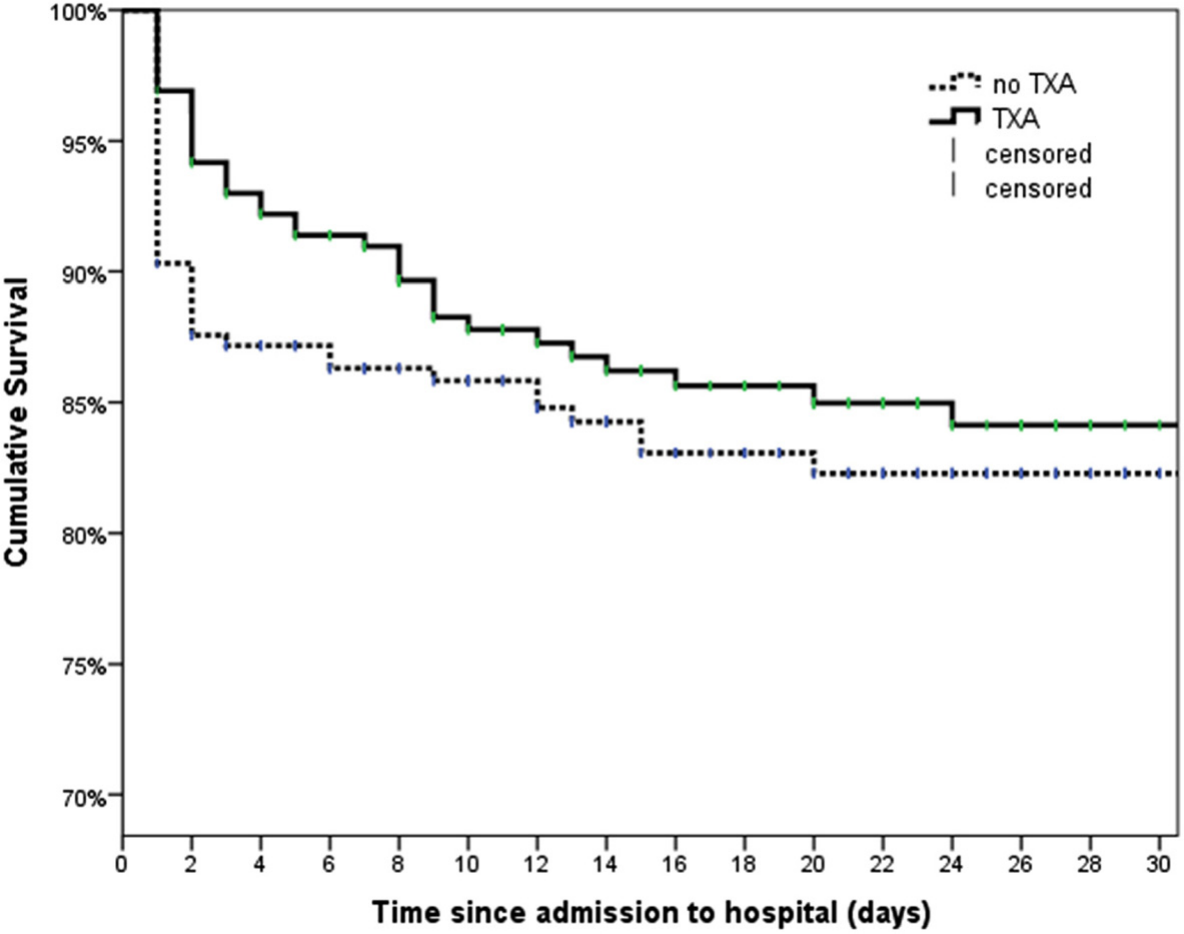
Kaplan-Meier survival rates up to 30 days following hospital admission. Data were censored in case of discharge or transfer. *p* = 0.472 (log-rank test). *TXA* tranexamic acid

Early mortality was significantly lower in the TXA group (e.g., 24-h mortality 5.8 % [TXA] vs. 12.4 % [control]; *p* = 0.01), and mean time to death was 8.8 ± 13.4 days (TXA) vs. 3.6 ± 4.9 days (control) (*p* = 0.001). Overall hospital mortality was similar in both groups (14.7 % vs. 16.3 %; *p* = 0.72). Correspondingly, these mortality rates are reflected in the survival curves, since for the initial days following ER arrival TXA patients tended to have higher survival (Fig. 2). The log-rank test yielded no significant differences for these Kaplan-Meier plots (*p* = 0.47).

**Fig. 3:**
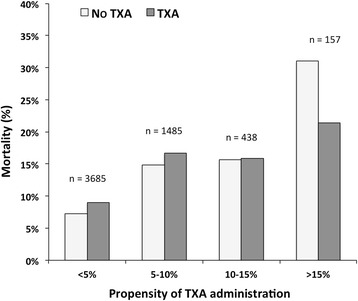
Mortality rates for patients with (*n* = 258) and without (*n* = 5507) TXA treatment in groups with increasing propensity of TXA administration (*p* < 0.001, χ^2^ test). Propensity of TXA administration was calculated by applying the predictors derived from Table 1. *TXA* tranexamic acid

In a subgroup analysis, Fig. 3 illustrates that the most pronounced decrease in mortality from TXA could be observed in those patients with a high propensity score, with the score indicating a more complicated prehospital course and higher injury severity as reflected by the factors derived from multivariate analysis shown in Table 1.

## Discussion

To our knowledge, this is the first civilian study to examine the effect of prehospital TXA use in trauma patients. Our propensity score-based matched-pairs analysis of a large population of trauma patients resulted in two identical groups, and the prehospital i.v. administration of TXA was associated with a significantly lower early mortality (up to 24 h) and increased time to death. Particularly those patients appeared to benefit most from TXA who had a high propensity score reflecting severe injury load. The improvement of early outcome may suggest benefits of TXA on haemostatic resuscitation, since previous investigations have indicated that the majority of deaths due to traumatic bleeding occur in the first few hours following trauma [19]. In this context, the recent Pragmatic Randomized Optimal Platelet and Plasma Ratios trial showed that exsanguination was the predominant cause of death within the first 24 h [5].

However, although the amount of blood components was lower in the TXA group, the difference was not significant. Surprisingly, only about 25 % in both groups required any transfusion at all. The lower mortality rates may also be explained by the anti-inflammatory effects of TXA [20], although further studies are required to elucidate this potential additional benefit. Multiple randomised controlled trials from elective surgery (e.g., orthopaedic, cardiovascular) have shown beneficial effects of TXA with respect to reduced perioperative blood loss [21]. Still, to date, CRASH-2 represents the only randomised controlled trial on TXA in trauma [8]. The researchers in that study recruited 20,211 trauma patients following hospital admission and showed that treatment with TXA significantly reduced all-cause mortality vs. placebo (14.5 % vs. 16.0 %). Death caused by haemorrhage was reduced as well (4.9 % vs. 5.7 %), without increasing thromboembolic events. The authors of a post hoc analysis observed beneficial effects, especially when TXA was given early (within 1 h after trauma), and delayed administration was even linked with worse outcomes [9]. However, several weaknesses and gaps of CRASH-2 have been discussed controversially [22]. First, data on laboratory values, on injury severity or on subtypes of transfused blood components (pRBC, fresh frozen plasma) were not reported. Second, the majority of patients were enrolled in low-income or developing countries, causing hesitancy to translate the findings to mature trauma systems. Retrospective military studies (Military Application of Tranexamic Acid in Trauma Emergency Resuscitation [MATTERs]) from a combat treatment facility in southern Afghanistan confirmed the benefits of TXA in battle casualties [23, 24]. The evidence from developed countries remains equivocal. In a single-centre retrospective analysis done in Miami, FL, USA, TXA was administered at a median of 97 minutes following ER admission to 150 patients deemed to be at high risk for haemorrhagic death. Propensity score-based matching demonstrated higher mortality in the TXA group [25]. In another single-centre study, done in London, UK, the majority of patients (*n* = 160) received TXA in the ER during the adoption phase of a major haemorrhage protocol [26]. However, this cohort was also more severely injured and more coagulopathic than non-TXA patients, since TXA was administered when critical injury or signs of haemorrhagic shock were present. Still, multivariate adjustments suggested potential benefits in severely injured shock patients. Furthermore, Moore et al. demonstrated that only a minority of severely injured patients presented with hyperfibrinolysis upon admission and a majority even displayed shut-down of fibrinolysis, raising concerns that TXA might cause complications in these patients [7]. Although CRASH-2 showed no difference with respect to vascular occlusive events, the authors admitted that, owing to trial design, they “might have underreported the frequency of these events” [8]. In the MATTERs study, TXA was associated with higher rates of thromboembolic events [23]. In our study, we found no difference, but data were incomplete. However, longer ICU stay and longer hospital stay in our TXA group might indicate a more complicated course.

The decision of several ADAC air rescue bases against TXA use reflects both the ongoing controversy regarding TXA and the uncertainty with applying a substance that has such weak evidence in the prehospital phase. In a recent review, Ausset et al. summarized that the evidence for the prehospital use of TXA regarding trauma populations is lacking [27]. Several groups have simply published their experience with the feasibility and frequency of prehospital TXA administration, but without reporting any outcome data [28–31].

Several limitations of the present study must be addressed. First, laboratory parameters with respect to hyperfibrinolysis or inflammation (e.g., d-dimers, thromboelastometry, interleukin-6) are not available in the databases. Second, the documentation of data was incomplete and inconsistent with respect to morbidity (organ failure, sepsis, thromboembolism). Furthermore, as the two databases were merged, only 5801 of 17,584 patients in the ADAC Air Rescue Service database could be assigned to a case in the TR-DGU. The data are further limited because exact timing of prehospital TXA administration and dosages (TXA or fibrinogen concentrate) have not been documented. Personal correspondence with all participating Air Rescue bases revealed that the majority apply a single dose of 1 g of TXA during prehospital treatment. Patients were treated with TXA at the discretion of the emergency physician, not according to a standardized algorithm, representing another major weakness. In this context, base excess and overall transfusion rates were moderate, which might indicate that TXA was also administered to noncoagulopathic patients or patients without signs of active bleeding. Also, the subtype of colloid infusion is not specified in the databases. A further limitation is that the cause of death (e.g., bleeding, brain death) is not documented in the TR-DGU; therefore, we cannot discern whether TXA was associated with reduced mortality due to haemorrhage.

Nevertheless, the present analysis represents the largest civilian study on prehospital TXA supplementation in trauma patients. The current European guidelines for management of bleeding and coagulopathy following major trauma recommend considering the administration of the first dose of TXA en route to the hospital. Since this is based on very limited evidence and extrapolated from the CRASH-2 trial, the authors graded this as a “very weak recommendation” (grade 2C) [10]. However, until further evidence emerges from ongoing prospective randomised controlled trials [32, 33], considering the existing literature on in-hospital administration, the results of the present study support the prehospital use of TXA in those trauma patients with severe injury with confirmed or suspected haemorrhagic shock.

## Conclusions

In the present study of trauma patients, prehospital use of TXA was associated with prolonged time to death and significantly improved early survival, suggesting benefits of TXA on haemostatic resuscitation. Until further evidence emerges, the results support the use of TXA during prehospital treatment of severely injured patients.

## Key messages

- Evidence from mature trauma systems on prehospital administration of TXA in civilian populations is not available.
- Prehospital use of TXA was associated with significantly longer time to death and significantly lower mortality rates at 6, 12 and 24 h.
- The most pronounced difference in mortality could be observed in those patients with a high propensity score reflecting severe injury load.
- The present data support the current recommendation to consider the first dose of TXA en route to the hospital.

## Abbreviations

ADAC: General German Automobile Club
AIS: Abbreviated Injury Scale
CPR: cardiopulmonary resuscitation
CRASH-2: Clinical Randomisation of an Antifibrinolytic in Significant Haemorrhage 2
ER: emergency room
FFP: fresh frozen plasma
GCS: Glasgow Coma Scale
DGU: Deutsche Gesellschaft für Unfallchirurgie
i.v.: intravenous
ICU: intensive care unit
INR: international normalized ratio
ISS: Injury Severity Score
LOS: length of stay
MATTERs: Military Application of Tranexamic Acid in Trauma Emergency Resuscitation
NACA: National Advisory Committee for Aeronautics
pRBC: packed red blood cells
PROPPR: Pragmatic Randomized Optimal Platelet and Plasma Ratios
PTI: prothrombin time index
RISC: Revised Injury Severity Classification
SBP: systolic blood pressure
SD: standard deviation
TBI: traumatic brain injury
TR: TraumaRegister
TXA: tranexamic acid
